# The impact of Yohimbine-induced arousal on facets of behavioural impulsivity

**DOI:** 10.1007/s00213-018-5160-9

**Published:** 2019-01-11

**Authors:** Aleksandra M. Herman, Hugo D. Critchley, Theodora Duka

**Affiliations:** 1 Behavioural and Clinical Neuroscience, School of Psychology, University of Sussex, Brighton, BN1 9QH UK; 2 Sussex Addiction and Intervention Centre, University of Sussex, Sussex, UK; 3 Sackler Centre for Consciousness Science, University of Sussex, Sussex, UK; 4 Department of Neuroscience, Brighton and Sussex Medical School, Sussex, UK; 5 Sussex Partnership NHS Foundation Trust, Sussex, UK

**Keywords:** Yohimbine, Noradrenaline, Heart rate, Blood pressure, Barratt Impulsiveness Scale, UPPS-P, Stop Signal Task

## Abstract

**Rationale:**

State-dependent changes in physiological arousal may influence impulsive behaviours.

**Objectives:**

To examine the relationship between arousal and impulsivity, we assessed the effects of yohimbine (an α_2_-adrenergic receptor antagonist, which increases physiological arousal via noradrenaline release) on performance on established laboratory-based impulsivity measures in healthy volunteers.

**Methods:**

Forty-three participants received a single dose of either yohimbine hydrochloride or placebo before completing a battery of impulsivity measures. Blood pressure and heart rate were monitored throughout the study.

**Results:**

Participants in the yohimbine group showed higher blood pressure and better response inhibition in the Stop Signal Task, relative to the placebo group. Additionally, individual changes in blood pressure were associated with performance on Delay Discounting and Information Sampling tasks: raised blood pressure following drug ingestion was associated with more far-sighted decisions in the Delay Discounting Task (lower temporal impulsivity) yet reduced information gathering in the Information Sampling Task (increased reflection impulsivity).

**Conclusions:**

These results support the notion that impulsive behaviour is dependent upon state physiological arousal; however, distinct facets of impulsivity are differentially affected by physiological changes.

**Electronic supplementary material:**

The online version of this article (10.1007/s00213-018-5160-9) contains supplementary material, which is available to authorized users.

## Introduction

Impulsivity describes a tendency to act rapidly without considering the consequences of one’s actions (Daruna and Barnes 1993; Moeller et al. 2001). The importance of this phenomenon is widely appreciated, both in everyday life, as it is a major influence on decision-making processes, and in the clinical expression of many neuropsychiatric conditions such as attention deficit and hyperactivity disorder (ADHD), manic episodes of bipolar disorder, Parkinson’s disease, eating disorders, or substance abuse (American Psychiatric Association 2013).

Impulsivity is a multidimensional construct, which can be considered both as a stable personality characteristic (trait) and as behaviour that varies depending on a situation (state impulsivity) (Herman et al. 2018a). Behavioural impulsivity can be divided into three major facets: reflection impulsivity (lack of information gathering and assessment before reaching a conclusion), motor impulsivity (premature or no longer appropriate actions) and temporal impulsivity (difficulty in delaying gratification) (reviewed in Herman et al. 2018a; Herman and Duka 2018). Additionally, inattention and aspects of disadvantageous decision-making, such as risk-taking, are sometimes also considered a part of impulsivity construct (for discussion, see de Wit 2009; Winstanley 2011; Herman et al. 2018a).

Current mood and/or arousal state (Herman et al. 2018a) is shown to induce changes in distinct facets of behavioural impulsivity. Arousal refers to the general level of cortical excitement and autonomic activation (Gray 1964) and ranges from drowsiness or sleep to behavioural activation or extreme emotional experience (Humphreys and Revelle 1984). Thus, increased arousal is intrinsic to high positive or negative emotional experience. Previous research indicates that modulating one’s state of physiological arousal causes changes in the performance on impulsivity tasks. For example, moderate physical exercise can decrease motor impulsivity (Chu et al. 2015). Furthermore, individual differences play a role in the relationship between state arousal and impulsivity. Individuals high in trait impulsivity typically have a low resting state of arousal (Fowles 2000; Mathias and Stanford 2003; Puttonen et al. 2008; Schmidt et al. 2013). Since every organism aims to reach an optimal internal state (i.e. one that feels best; Hebb 1955), it is hypothesised that these individuals behave impulsively in order to increase their arousal to the optimal level (Barratt 1985; Eysenck and Eysenck 1985; Zuckerman 1969). Thus, trait impulsivity, associated with a low resting state of arousal, might mediate the effects of arousal on behaviour. For instance, impulsive individuals perform worse at baseline than low-impulsive individuals on tests of sustained attention, but they obtain a greater performance benefit from caffeine than the low-impulsive individuals (Smith et al. 1991), suggesting that such a manipulation of (psycho) physiological state will also influence state impulsivity. This is further supported by clinical effects of treating ADHD patients with stimulant drugs: medications such as methylphenidate increase arousal levels and can decrease impulsive behaviour (Swanson et al. 2011). In healthy populations, low resting physiological arousal, reflected in low heart rate, predicts faster responses and riskier behaviour in a gambling game, indicating diminished impulse control (Schmidt et al. 2013). Interestingly, participants tend to make fewer risky gambles following physical exercise, when compared to a resting condition. Thus, while relationship between bodily arousal and impulsive behaviours might yield valuable insights for clinical practice, there has yet to be a comprehensive study that looks at how physiological arousal differentially affects dissociable aspects of impulsivity.

State arousal can be modulated pharmacologically with substances that act on the noradrenergic system. Yohimbine hydrochloride, an α_2_-adrenergic receptor antagonist, increases blood norepinephrine levels (Hedner et al. 1992) and causes an increase in physiological arousal (Goldberg et al. 1983; Krystal et al. 1992; Swann et al. 2013). At higher doses, yohimbine can induce hypertension, change mood state and increase anxiety and nervousness (Cimolai and Cimolai 2011) in particular in individuals more prone to anxiety (Gurguis et al. 1997). Thus, induced arousal appears to be a more common effect of yohimbine.

Evidence for the effects of yohimbine on impulsivity mostly comes from animal research. For example, yohimbine acutely increases the preference for the large and delayed reinforcer over a smaller immediate one (decreased temporal impulsivity) (Schippers et al. 2016). However, yohimbine might also induce inflexibility in adjusting behaviour in response to changes in the relative reward values of different response options (Schwager et al. 2014; Montes et al. 2015). Moreover, the behavioural effects of yohimbine might depend on individual differences: yohimbine improves response inhibition (i.e. decreases ‘stopping’ motor impulsivity) in highly impulsive rats but attenuates response inhibition in low-impulsive rats (Schippers et al. 2016). Yohimbine also induces dose-dependent increases in premature responding on the Five-Choice Serial Reaction Time Task (‘waiting’ motor impulsivity) in rats (Sun et al. 2010; Mahoney et al. 2016) and decreases attentional performance; however, the effects do not depend on baseline impulsivity levels (Barlow et al. 2018). In humans, yohimbine is shown to increase impulsive behaviour on the Immediate Memory Task (IMT) and Delayed Memory Task (DMT) (a measure of ‘waiting’ motor impulsivity), which correlates with increases in blood pressure (Swann et al. 2005, 2013), an effect often associated with arousal. To our knowledge, there are no studies in humans examining the effects of yohimbine on impulsivity using tasks to test the different facets of impulsivity simultaneously. And from the findings presented above, it also seems that yohimbine may have differential effects on distinct facets of impulsivity (i.e. decrease motor ‘stopping’ and temporal impulsivity but increase ‘waiting’ impulsivity and inattention) which may depend on baseline impulsivity levels.

Thus, the aims of the current study were twofold: firstly, to determine whether yohimbine differently affects the distinct facets of behavioural impulsivity. We hypothesised that yohimbine administration would lead to lower behavioural impulsivity, specifically motor ‘stopping’ and temporal subtypes, particularly in more impulsive individuals (Barratt 1985; Eysenck and Eysenck 1985; Zuckerman 1969). Alternatively, increasing noradrenergic activity may increase aspects of impulsive behaviour via deleterious effects on the scope of attention (Robbins 1997). Secondly, we explored the alleged under-arousal hypothesis of trait impulsivity. In line with past literature (Fowles 2000; Mathias and Stanford 2003; Puttonen et al. 2008; Schmidt et al. 2013), we predicted that more impulsive individuals would show lower resting levels of physiological arousal. We used two trait impulsivity measures: the Barratt Impulsiveness Scale and the UPPS Impulsivity Scale, to capture the wide range of impulsivity characteristics, including emotional impulsivity (positive and negative urgency).

Healthy volunteers took part in a double-blind study. Participants were randomly assigned to a control (placebo) or experimental (yohimbine) groups and completed a battery of behavioural impulsivity tasks. We compared performance of the two groups to test how noradrenergic manipulation influenced distinct domains of impulsive behaviour.

## Materials and methods

### Participants

The study design was approved by the BSMS Research Governance and Ethics Committee. Forty-three healthy volunteers (19 males) were randomly assigned to one of two experimental groups: placebo or yohimbine. Only volunteers who met strict inclusion criteria were recruited. These criteria involved the following: age between 18 and 40 years old, normal or corrected-to-normal vision, no lifetime history of any neurological or mental disorders, no current pharmacological treatment or psychological counselling, no drug use within 5 days prior the testing session or alcohol use 24 h before testing session, weight above 55 kg, systolic blood pressure (SYS BP) below 135 mmHg and diastolic blood pressure (DIA BP) below 90 mmHg. Strict exclusion criteria involved a history of anxiety or panic attacks. Women who were not using a recommended means of birth control undertook a pregnancy test before participation in the study. All volunteers gave written informed consent to participate and received compensation for their time (£10 per hour).

### Materials

#### Questionnaires

Each participant completed a battery of questionnaires to assess current mood state, alcohol use and impulsivity. The *Nuffield Hospitals Medical History Questionnaire* was used to record demographic details, past and present health status, use of medications and recreational drugs and a number of cigarettes smoked per day.

The *Barratt Impulsiveness Scale* (BIS-11) (Patton et al. 1995) and the *UPPS-P Questionnaire* (Whiteside and Lynam 2001; Cyders and Smith 2007), widely used questionnaires in impulsivity research, measured trait impulsivity. BIS provides an index of three impulsivity dimensions: motor, non-planning and in-attention. UPPS-P gives a measure of premeditation, perseverance, sensation seeking as well as tendencies to act impulsively while experiencing positive and negative emotions and positive and negative urgency, respectively.

Participants completed the *Rey Auditory Verbal Learning Test* (RAVLT; Rey 1964), a measure of working memory capacity, to ensure that both experimental groups are matched on the basis of their cognitive abilities. Participants heard a list of 15 unrelated nouns with a presentation rate of one word per 2 s. Following a period of 2 min, while instructed to count from 100 backwards out-loud to minimise mental repetition, participants were asked to recall as many words as they could remember. The number of correct recalls was the dependent variable.

The *Alcohol Use Questionnaire* (AUQ, Mehrabian and Russell 1978) provided an estimate of a number of alcohol units (1 unit = 8 g of alcohol) consumed a week over the past 6 months.

The *Depression Anxiety Stress Scale* (DASS; Henry and Crawford 2005) consists of three seven-item self-report scales that measure the extent of depression, anxiety and stress experienced over the past week. This scale was introduced to ensure group matching on negative mood ratings.

The *Drug Effects Questionnaire* (DEQ; Morean et al. 2013) assesses two key aspects of subjective experience: the strength of substance effects and the desirability of substance effects. It consists of five items, “Do you feel a drug effect right now?” (feel), “Are you high right now?” (high), “Do you like any of the effects you are feeling right now?” (like), “Do you dislike any of the effects you are feeling right now?” (dislike) and “Would you like more of the drug you took, right now?” (more), rated on a 100-point visual analogue scale ranging from “not at all” to “extremely”.

The *Perceived Arousal Scale* (Anderson et al. 1995) provides ratings of subjective arousal state. It consists of 24 adjectives indicating arousal (e.g. energetic) or a lack of arousal (e.g. sleepy) rated on a five-point scale from 1 (“very slightly or not at all”) to 5 (“extremely”). The scale has a high internal consistency (Cronbach’s *α* = .93).

The *Positive Affect/Negative Affect Scale* (PANAS) (Watson et al. 1988) is a 20-item measure of self-reported positive affect (PA) and negative affect (NA) experienced at the present moment.

The State-Trait Anxiety Inventory (STAI; Spielberger et al. 1983) was used to assess anxiety levels. It consists of two 20-item scales rated on a four-point scale.

#### Tasks

The *Affective Stop Signal Task* (ASST) measured motor response inhibition in task-irrelevant emotional contexts. This modified version of the commonly used Stop Signal Task was introduced as previous reports suggested that yohimbine might affect amygdala responses to fearful faces and change the perception of emotional faces (Schwabe et al. 2013). Therefore, we used a paradigm with task-irrelevant emotional context (fearful faces).

The details on the ASST were published previously (Herman et al. 2018b). Briefly, instead of arrows, participants were presented with facial expressions from the FACES database (Ebner et al. 2010) of males and females (50% each) displaying either fear or neutral expression (50% each). On the Go-trials (a facial expression surrounded by a white frame), participants were instructed to respond with an appropriate button press to indicate whether the face displayed on the screen was male or female (implicit emotional context) as quickly as possible and to try and withhold their responses when the frame surrounding the picture changed colour (Stop-trials). The onset of the Stop Stimulus (the same picture surrounded by a yellow frame) was adjusted according to a staircase procedure depending on individual performance separately for each emotional condition, to obtain a probability of stopping of 0.5 for each condition. Participants were informed that speed and accuracy on the task are equally important and that they should not be delaying their responses to see whether the frame would turn yellow. The Stop-Signal Reaction Time (SSRT) was calculated separately for neutral (SSRT Neutral) and fearful (SSRT Fearful) trials.

Participants completed two runs of 160 trials with a rest break in between. In total, there were 120 Go Neutral, 120 Go Fearful, 40 Stop Neutral and 40 Stop Fearful trials.

The *Probability Discounting Task* (PD; Madden et al. 2009) is a measure of risk-taking. It consists of a list of 30 choices between smaller certain rewards and uncertain larger gains. The dependent variable is *h* parameter calculated for each participant using the following formula: *h* = (Probabilistic reward / Certain reward − 1) / Odds against winning) (ln-transformed to reduce skewness). Large *h* values indicate discounting of probabilistic rewards (risk aversion).

The *Information Sampling Task* (IST; Clark et al. 2006) is a measure of reflection impulsivity. On each trial, a matrix of 5 × 5 grey squares was presented on a computer screen. The participant selected a square by clicking with the mouse over the square, to reveal one of two colours (e.g. red and blue) until they were confident which of the two colours was in the majority of the squares. There were two conditions of the task:

(i) IST fixed win condition (FW): the participant won 100 points if they made the right decision (regardless of how many boxes they have opened); otherwise, they lost 100 points. The participant completed 10 experimental trials.
(ii) IST reward conflict (RC): for every box opened, the participant lost 10 points from a bank of 250. If the participant chose correctly, they won the remaining points from the bank; otherwise, they lost 100 points. Each participant completed 10 experimental trials.

The dependent variable for both conditions is *p* (correct), which reflects the degree of certainty that a participant requires when they make a decision. *p* (correct) values of 1 indicate that the participant acquired full information before deciding, and 0.5 indicates that the participant had only enough information to choose at chance.

The *Monetary Choice Questionnaire* (MCQ; Kirby et al. 1999) is a measure of temporal impulsivity. Each participant was presented with 27 hypothetical choices between small and immediate rewards (SIR) and larger delayed rewards (LDR), for example “would you prefer £54 today or £55 in 117 days?”. The dependent variable was the proportion of LDR choices made.

### Procedures

Before testing, all volunteers attended a standardised interview with a medical doctor (TD). The screening checked for exclusion criteria, history of medication and recreational drug use, contraceptive use, any current or chronic medical condition and current or lifetime history of any psychiatric or neurological disorder. Seventy-four volunteers (48 females) entered initial screening, but 27 (36%) were excluded as they met one or more exclusion criteria, and further four individuals (5%) withdrew from the study, yielding 43 individuals who participated.

Participants were instructed to refrain from caffeine-containing products on a day of testing and have a light breakfast in the morning before participating in the study. Following completion of RAVLT, Alcohol Use and PANAS questionnaires and BP measurement, participants were administered 20 mg yohimbine (yohimbine hydrochloride; Arzneimittel GmbH) or placebo orally 45 min before the behavioural testing began. Sample size, dosage and timing of drug administration were chosen according to previous studies using yohimbine, as this dosage was shown to evoke mild effects on the physiological arousal without causing mood-related side effects (anxiety and nervousness) (Plewnia et al. 2001; Schwabe et al. 2010, 2012, 2013; Swann et al. 2013).

**Fig. 1:**
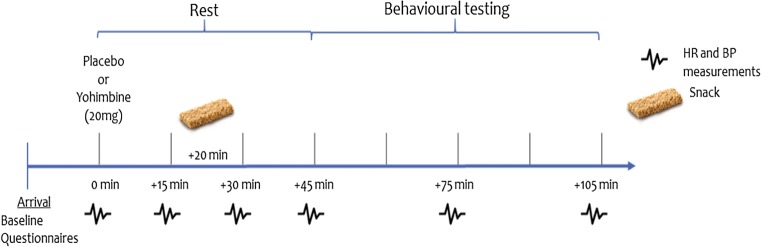
Procedures and timeline during the testing session

Within the first 45 min following tablet administration, the participants had time to relax and their heart rate (HR) and BP was monitored every 15 min. Subsequently, physiological measurements were taken every 30 min. All physiological measures were recorded while participants were sitting still. We report both systolic and diastolic effects on blood pressure as per previous literature to mitigate confounding differential effect of the drug on DIA and SYS BP (e.g. Krystal et al. 1992; Swann et al. 2013). Approximately 20 min following the tablet administration, a light snack was served. Following a 45-min rest period, testing part commenced, during which participants completed behavioural impulsivity measures (ASST, IST, PD and MCQ, in a randomised order) and further state measure questionnaires (PANAS, Perceived Arousal Scale and DEQ). Procedures are illustrated in Fig. 1. After behavioural testing was completed, participants remained in the lab until their BP was < 10 mmHg above baseline.

### Statistical analysis

An exploratory correlational analysis was undertaken to assess the relationship between the resting level of physiological arousal (HR, BP) and trait impulsivity measures. Differences between groups on demographic information and task performance (apart from the ASST) were compared using a series of independent samples *t* tests or chi-square tests as appropriate. Response inhibition on the ASST was analysed with mixed ANOVA with emotion condition (fearful and neutral) as a within-subjects factor, and group (yohimbine or placebo) as a between-subjects factor. Physiological measures were also analysed using mixed ANOVAs with time of measurement as a within-subjects factor, and group (yohimbine or placebo) as a between-subjects factor. Significant main or interaction effects were pursued with appropriate follow-up tests, including repeated-measures ANOVA. In case of violation of sphericity, multivariate ANOVAs were used (Maxwell and Delaney 2004). Additionally, for completeness, in the Appendix in the Electronic supplementary material, we also present the analysis exploring sex differences in questionnaire and behavioural data.

## Results

### Exclusions and missing data

One participant did not complete the study due to strong nausea and cardiovascular reaction to yohimbine. Therefore, the final sample consisted of 42 participants (23 females), of which 21 (12 females) received placebo and 21 (11 females) yohimbine. The number of cigarettes per smoker in each group was similar (3 and 4, respectively; *X*^2^(1) = 0.17, *p* = .679). Five participants did not complete RAVLT, and data from MCQ were missing for two individuals, due to technical failure; all data from questionnaires administered after tablet ingestion (PANAS, Perceived Arousal Scale and DEQ) were missing for one participant. Four participants were excluded from the ASST for failing to follow instructions not to wait for the stop signal, evidenced by long Go RTs, long SOA values and/or high Stop Accuracy values (> 2.5 standard deviations from the group mean).

The groups were well matched on demographics, mood state and personality variables. However, there were some group differences in sensation seeking (not significant after the Bonferroni correction for multiple comparisons, *p* > .003) (see Table 1). Therefore, to investigate the potential role of sensation seeking, each comparison was computed with and without including sensation seeking as a confounding covariate.

**Table 1:** Group demographics, personality and mood state measures as well as group statistics

Variable	Placebo			Yohimbine			*t*	*df*	*p*	Cohen’s *d*	95% CI	
	*N*	Mean	SD	*N*	Mean	SD					Lower	Upper
Demographic information												
Age	21	21.29	3.27	21	23.19	5.41	− 1.38	40	.175	− 0.43	− 4.69	0.88
Weight (kg)	21	70.99	10.72	21	68.96	8.15	0.69	40	.493	0.21	− 3.9	7.97
Height (m)	21	1.76	0.1	21	1.73	0.09	0.84	40	.406	0.26	− 0.04	0.08
BMI (kg/m^2^)	21	22.96	2.68	21	22.97	2.37	− 0.02	40	.988	− 0.01	− 1.59	1.57
Alcohol units per week	21	12.91	10.5	21	11.4	11.55	0.45	40	.659	0.14	− 5.37	8.4
RAVLT	18	6.56	2.12	19	5.9	1.45	1.11	35	.274	0.37	− 0.55	1.87
Trait impulsivity												
BIS total	21	66.95	10.52	21	62.76	9.93	1.33	40	.192	0.41	− 2.19	10.57
UPPS-P												
Negative urgency	21	28.91	6.58	21	25.19	5.4	2	40	.052	0.62	− 0.04	7.47
Premeditation	21	22.38	4.57	21	19.95	5.2	1.61	40	.116	0.5	− 0.62	5.48
Perseverance	21	21.05	4.93	21	18.81	5.22	1.43	40	.161	0.44	− 0.93	5.41
Sensation seeking	21	40.24	5.21	21	34.57	7.53	2.83	40	*.007*	0.88	1.63	9.71
Positive urgency	21	30.43	10.19	21	26.52	7.31	1.43	40	.161	0.44	− 1.63	9.44
Mood measures												
PANAS												
NA pre	21	11.71	2.43	21	12.86	2.46	− 1.52	40	.138	− 0.47	− 2.67	0.38
PA pre	21	28.33	5.8	21	30.38	7.48	− 0.99	40	.327	− 0.31	− 6.22	2.13
STAI												
Trait anxiety	21	39.1	7.08	21	39.71	7.46	− 0.28	40	.784	− 0.09	− 5.15	3.92
State anxiety	21	34.91	7.88	21	32.71	7.81	0.91	40	.371	0.28	− 2.7	7.08

### Blinding

**Fig. 2:**
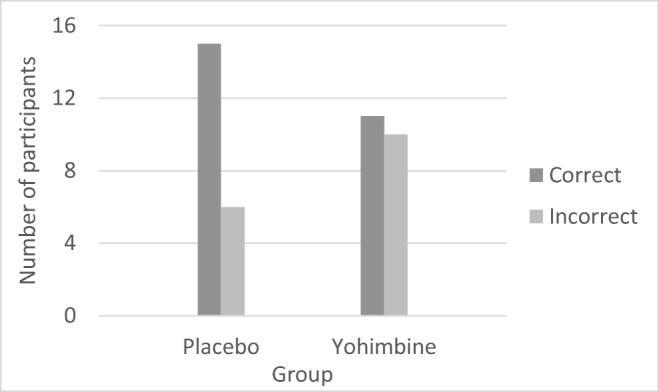
Deception-individuals’ insights into the group allocation

To establish whether the blinding procedure was successful, we compared the numbers of participants who correctly and incorrectly guessed their group allocation. Chi-square test was insignificant (*X*^2^(1) = 1.62, *p* = .204), indicating that individuals in both the placebo and yohimbine groups were blind to the group allocation. Notably, 11 out of 21 participants in the yohimbine group thought they received placebo, while 15 out of 21 participants from the placebo group thought they received placebo; therefore, the blinding procedure seemed to work marginally better for the yohimbine group (see Fig. 2 for details).

### Resting state arousal and trait impulsivity

Correlational analysis to examine the relationship between resting state measures of arousal (HR, DIA BP, SYS BP) and trait impulsivity measures (BIS and UPPS-P) showed no significant correlations (Table 2), indicating that, in this group, trait impulsivity was not related to unusually low levels of arousal at rest.

**Table 2:** Pearson’s correlations between trait impulsivity dimensions and measures of physiological arousal at baseline

	Baseline BP SYS (mmHg)	Baseline BP DIA (mmHg)	Baseline HR (bmp)
BIS total score			
Pearson’s *r*	.039	.234	− .146
*p* value	.806	.136	.356
UPPS-P			
Premeditation			
Pearson’s *r*	.109	.292	− .102
*p* value	.493	.060	.521
Perseverance			
Pearson’s *r*	.056	.107	.034
*p* value	.723	.500	.829
Sensation seeking			
Pearson’s *r*	.074	.051	− .245
*p* value	.642	.750	.118
Negative urgency			
Pearson’s *r*	.089	.071	− .059
*p* value	.575	.653	.709
Positive urgency			
Pearson’s *r*	.292	.259	− .182
*p* value	.061	.098	.250

### Yohimbine effects on affective state

Following drug ingestion, the yohimbine group reported increased levels of NA but did not differ from the placebo group in PA (Table 3). No group differences in self-perceived arousal or drug effects were found. The results did not change after including sensation seeking as a covariate.

**Table 3:** Descriptive statistics of the mood state measures as well as task performance group comparison following drug/placebo ingestion

Variable	Placebo			Yohimbine			*t*	*df*	*p*	Cohen’s *d*	95% confidence interval	
	*N*	Mean	SD	*N*	Mean	SD					Lower	Upper
State questionnaires												
DEQ												
Feel	20	27.9	26.49	21	28.19	28.79	− 0.03	39	0.973	− 0.01	− 17.79	17.21
High	20	20.15	22.76	21	15.43	24.3	0.64	39	0.525	0.2	− 10.17	19.61
Dislike	20	21.85	27.46	21	22.29	24.01	− 0.05	39	0.957	− 0.02	− 16.71	15.84
Like	20	34.6	21.23	21	39.52	27.37	− 0.64	39	0.525	− 0.2	− 20.45	10.6
Want more	20	27.85	22.1	21	21.81	24.69	0.82	39	0.415	0.26	− 8.79	20.87
Perceived arousal	20	69.65	21.19	21	79.62	17.88	− 1.63	39	0.111	− 0.51	− 22.33	2.39
PANAS												
PA post	20	21.3	8.42	21	26.29	9.72	− 1.75	39	0.088	− 0.55	− 10.74	0.77
NA post	20	10.85	1.27	21	13.48	4.14	− 2.72	39	*0.010*	− 0.85	− 4.58	− 0.67
Task performance												
IST												
FW P (correct)	21	0.8	0.09	21	0.81	0.12	− 0.29	40	0.773	− 0.09	− 0.07	0.05
RC P (correct)	21	0.73	0.06	21	0.72	0.1	0.6	40	0.552	0.19	− 0.04	0.07
MCQ												
Proportion LDR	19	0.43	0.16	21	0.49	0.22	− 1.02	38	0.315	− 0.32	− 0.19	0.06
PD												
ln *h*	21	2.83	3.79	21	2.21	1.64	0.68	40	0.499	0.21	− 1.21	2.44
ASST												
SSRT neutral	19	293.47	58.79	19	280.34	41.52						
SSRT fearful	19	313.94	67.96	19	279.9	38.15						

### Yohimbine effects on physiological recordings

#### Systolic blood pressure

**Fig. 3:**
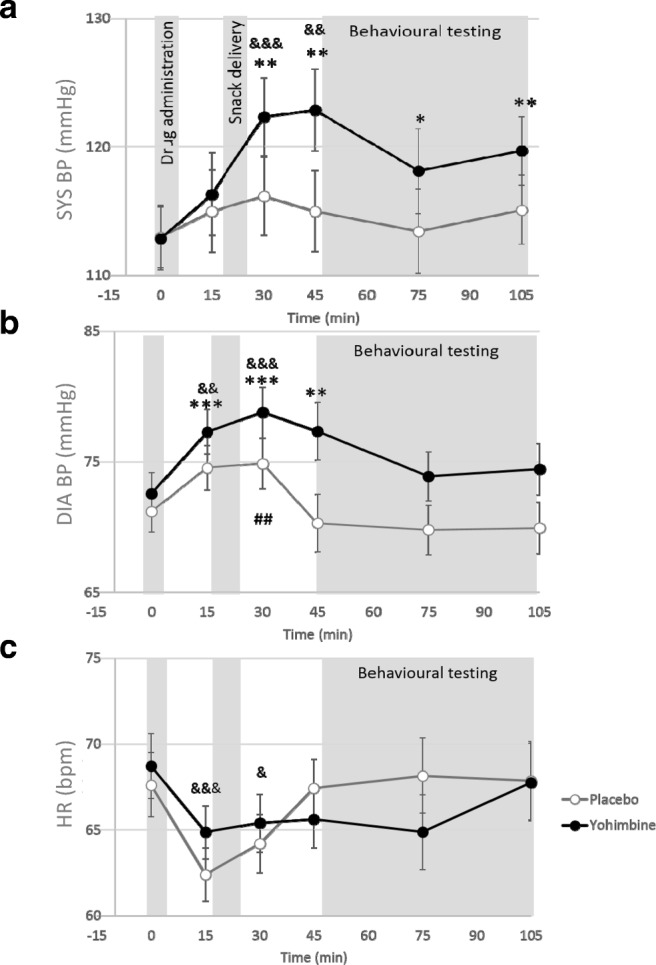
Measurements of **a** systolic blood pressure, **b** diastolic blood pressure and **c** heart rate of the yohimbine and placebo groups across the session. Error bars represent standard error. Significant difference from baseline: *p < .05, **p < .01, ***p < .001 (yohimbine group); ^##^*p* < .01 (placebo group); ^&^*p* < .05, ^&&^*p* < .01, ^&&&^*p* < .001 (main effect of time)

Mixed ANOVA revealed a trend for a time-group interaction (*F*(5, 200) = 1.90, *p* = .096, *η*^2^_p_ = .045) and a significant main effect of time (*F*(5, 200) = 4.81, *p* < .001, *η*^2^_p_ = .107), and no main effect of drug (*F*(1, 40) = 1.19, *p* = .28, *η*^2^_p_ = .029). The SYS BP reached its peak 45 min following drug administration (see Fig. 3a). Including SS as a covariate strengthened the interaction effect (*F*(5, 195) = 2.62, *p* = .026, *η*^2^_p_ = .063), and the main effect of time was no longer significant *F*(5, 195) = 0.712, *p* = .615, *η*^2^_p_ = .018). Post hoc repeated-measures ANOVA revealed that while the placebo group did not show significant changes in SYS BP over time (*F*(5, 100) = 1.18, *p* = .326), the yohimbine group did show changes over time (*F*(5, 100) = 4.10, *p* = .002).

#### Diastolic blood pressure

Mauchly’s test of sphericity was significant (*X*(14) = 39.41, *p* < .001); therefore, multivariate test was used. Wilks’ lambda test revealed a time-group interaction (*F*(5, 36) = 2.63, *p* = .040, *η*^2^_p_ = .267) and a main effect of time (*F*(5, 36) = 7.77, *p* < .001, *η*^2^_p_ = .519). Post hoc tests revealed that both groups showed DIA BP changes over time (placebo: *F*(5, 100) = 4.49, *p* = .001; yohimbine: *F*(5, 100) = 4.66, *p* = .001). The SYS BP reached its peak 30 min following drug administration (see Fig. 3b). There was a trend for a main drug effect (*F*(1, 40) = 3.04, *p* = .086, *η*^2^_p_ = .071), suggesting an overall higher DIA BP in the yohimbine group regardless of the time of measurement.

After controlling for SS, the interaction effect was approaching significance (*F*(5, 35) = 2.47, *p* = .051, *η*^2^_p_ = .261), the main effect of time was no longer significant (*F*(5, 35) = 0.893, *p* = .496, *η*^2^_p_ = .113) and the main effect of drug remained unchanged (*F*(1, 40) = 3.22, *p* = .080, *η*^2^_p_ = .076).

#### Heart rate

There was a trend for a time-condition interaction (*F*(5, 200) = 1.95, *p* = .088, *η*^2^_p_ = .05) and a main effect of time (*F*(5, 195) = 5.24, *p* < .001, *η*^2^_p_ = .12), but not a main effect of drug (*F*(1, 40) = 0.001, *p* = .975, *η*^2^_p_ = .00) (see Fig. 3c).

After controlling for SS, there were no significant results (interaction term: *F*(5, 195) = 1.43, *p* = .216, *η*^2^_p_ = .04; time: *F*(5, 195) = 0.62, *p* = .687, *η*^2^_p_ = .02; drug: *F*(1, 39) = 0.26, *p* = .611, *η*^2^_p_ = .01). Therefore, yohimbine did not affect the HR.

### Performance on the tasks

#### ASST

**Fig. 4:**
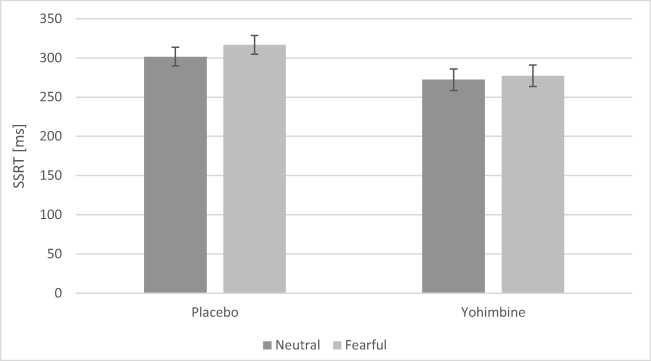
Group performance on the ASST. Results presented after controlling for sensation seeking

A main effect of drug (*F*(1, 35) = 4.30, *p* = .045, *η*^2^_p_ = .11) but not main effect of emotion or drug-emotion interaction effect (*p*s > .05) was found in the SSRT, indicating that under yohimbine participants had lower SSRT (i.e. they were better able to inhibit prepotent motor responses successfully). This effect, however, was only significant when controlling for individual differences in SS (Fig. 4).

#### MCQ, PD and IST

Independent samples *t* test revealed that there were no group differences in performance on neither MCQ, PD nor IST, and controlling for SS had no effects on the results (Table 3). Therefore, yohimbine ingestion did not affect temporal impulsivity, or risk-taking, or reflection impulsivity.

#### Correlations

**Fig. 5:**
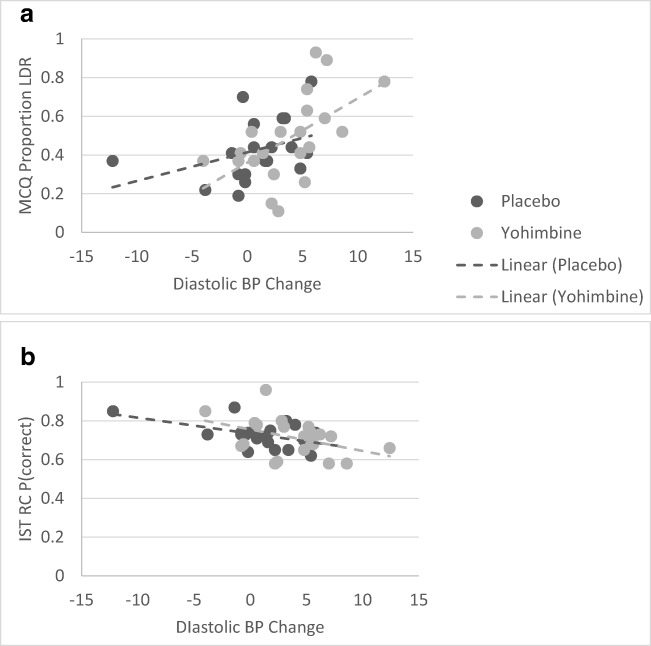
Scatterplots showing the relationship between the change in diastolic blood pressure and the proportion of larger delayed rewards (LDR) selected in the MCQ task (**a**) and the level of information gathering in the IST reward conflict condition (**b**). Different shades of grey depict the yohimbine and placebo groups

To further explore the relationship between individual changes in arousal and performance on the tasks, bivariate correlation coefficients were computed between post-drug ingestion changes in physiological parameters (BP and HR) and task-dependent variables. In this analysis, for each participant, we subtracted the baseline measurement from the average of post-tablet administration arousal measurements. Therefore, the change in arousal reflected increased state arousal following tablet ingestion relative to baseline level. The Bonferroni correction for multiple comparisons was set to *p* < .006. The results indicated that increased DIA BP was associated with a higher proportion of delayed versus immediate rewards chosen in the MCQ (Table 4, Fig. 5a). Elevated DIA BP and SYS BP were also associated with less impulsive responding in the fearful context in the ASST, but this correlation did not survive the correction for multiple comparisons. In contrast, elevated DIA BP was associated with less information sampling in the IST RC condition (increased reflection impulsivity; Fig. 5b). There were no other significant correlations (Table 4). To establish whether the relationships are solely related to changes in physiological arousal and not mood state, we additionally computed correlations between behavioural impulsivity measures and change in PANAS scores. There were no significant correlations (Table 4), indicating that state impulsivity level was solely driven by changes in physiological arousal level.

**Table 4:** Correlations between changes in physiological and mood state parameters (delta = average post-drug measurement − pre-drug measurement) and performance on the impulsivity tasks

Pearson correlations	DIA BP delta	SYS BP delta	HR delta	PANAS PA delta	PANAS NA delta
SSRTN					
Pearson’s *r*	0.182	− 0.177	0.039	0.084	− 0.182
*p* value	0.275	0.288	0.818	0.623	0.282
*N*	38	38	38	38	38
SSRTF					
Pearson’s *r*	− 0.371*	− 0.335*	0.021	− 0.010	− 0.015
*p* value	0.022	0.040	0.899	0.953	0.932
*N*	38	38	38	38	38
IST FW P (correct)					
Pearson’s *r*	− 0.091	0.065	0.174	− 0.037	0.211
*p* value	0.564	0.682	0.271	0.820	0.185
*N*	42	42	42	42	42
IST RC P (correct)					
Pearson’s *r*	− 0.444**	− 0.214	− 0.141	− 0.281	− 0.079
*p* value	*0.003*	0.173	0.372	0.075	0.622
*N*	42	42	42	42	42
MCQ proportion LDR					
Pearson’s *r*	0.496**	− 0.036	0.006	− 0.185	0.285
*p* value	*0.001*	0.826	0.969	0.259	0.078
*N*	40	40	40	40	40
PD ln *h*					
Pearson’s *r*	− 0.098	0.056	0.112	− 0.117	− 0.176
*p* value	0.536	0.724	0.480	0.466	0.271
*N*	42	42	42	42	42

Values in italics depict correlations that survived the Bonferroni correction for multiple comparisons (*p* < .006)

*p < .05 (uncorrected); **p < .01 (uncorrected)

## Discussion

The current study examined the role of state arousal induced by administration of α_2_-noradrenergic blocker, yohimbine, on distinct subtypes of behavioural impulsivity. We hypothesised that yohimbine-induced arousal would result in decreased impulsive behaviour.

In agreement with previous reports, yohimbine did not affect HR but caused an increase in BP, notably DIA BP (Krystal et al. 1992; Swann et al. 2005, 2013; Schwabe et al. 2010), proving to be a successful method of arousal induction, nevertheless indicating suppression of the baroreflex where increased blood pressure is associated with cardiac slowing. Moreover, the yohimbine group presented increased negative affective state ratings relative to placebo, with no differences in positive affective state ratings. This is consistent with previous findings indicating anxiogenic effects of yohimbine (Mattila et al. 1988; Cameron et al. 1994; Cimolai and Cimolai 2011; Elman et al. 2012; Moran-Santa Maria et al. 2014).

The yohimbine group outperformed the placebo group at response inhibition in the ASST, as predicted. There were no group differences in performance in either risk-taking, or reflection, or temporal impulsivity tasks. However, increased arousal, indexed by heightened DIA BP following drug administration, was associated with less impulsive behaviour in the MCQ (temporal impulsivity) and marginally the ASST (motor impulsivity) tasks, albeit regardless of the emotional context, but more impulsive behaviour on the IST RC task (increased reflection impulsivity). Additionally, the behavioural performance was associated with the changes in blood pressure only, and not self-reported mood differences, indicating that an increase in physiological arousal is driving the effects. However, no association between trait impulsivity and resting state arousal was found; thus, the findings provide only partial support for our hypotheses.

### Motor impulsivity

The yohimbine group showed lower motor impulsivity than placebo in the ASST, regardless of emotional context. This relationship, however, was only present when we controlled for individual differences in sensation seeking, indicating that personality characteristics might be an important factor for the role of arousal in inhibitory control. It seems vital to note that in one study, sensation seeking correlated with performance on the Stop Signal Task (Muhlert et al. 2015), suggesting that sensation seeking might play an important role in motor inhibition.

Overall, the findings of decreased motor impulsivity in the yohimbine group, which showed an increased level of arousal, and the correlational results of increased DIA BP following the drug administration versus baseline linked to better response inhibition (although this relationship did not survive the correction for multiple comparisons), in the fearful context, support our hypothesis of reduced motor impulsivity in a state of heightened physiological arousal. These results also corroborate previous findings. For example, abrupt alerting cues (i.e. an irrelevant external signal that appears briefly), which temporarily increase psychophysiological arousal (i.e. phasic alertness), were found to improve the ability to stop an already initiated response (Weinbach et al. 2015). Similarly, response inhibition capacity seems to be affected by acute changes in cardiovascular arousal state within the cardiac cycle, such that participants are more likely to successfully inhibit motor responses during cardiac systole (increased state of physiological arousal) than diastole (lower state of cardiac arousal) (Rae et al. 2018). SSRT, an index of difficulty at motor response inhibition, also decreases after acute exercise (Joyce et al. 2009; Chu et al. 2015). Moreover, atipamezole, another antagonist of ɑ_2_-adrenergic receptors, decreases SSRT in a rodent version of Stop Signal Task (Bari and Robbins 2013). Taking the previously reported data and our findings together, we conclude that a moderate increase in the level of arousal is related to a decrease in motor ‘stopping’ impulsivity.

However, we observed no group differences in motor impulsivity in the neutral and fearful conditions on the ASST. This may suggest that putative yohimbine-induced changes in the processing of emotional faces (Schwabe et al. 2013) may not be interfering with response inhibition capacities. However, this may be partly attributable to possible sex differences associated with yohimbine-induced effects on emotional processing (Schwabe et al. 2013). The sample sizes of our study were not powered to disentangle these effects reliably (however, see the Appendix in the Electronic supplementary material for details). Future studies should address this issue.

### Temporal impulsivity

Although we did not observe any group differences in performance in any other tasks apart from ASST, we found associations between post-drug administration arousal change and impulsive decisions. Specifically, increased DIA BP following drug administration was associated with fewer impulsive choices in the MCQ task, suggesting that increased arousal at subject level was associated with lower temporal impulsivity. Previous studies examining the relationship between physiological arousal and delay discounting mainly studied stress reactivity: the reported findings are mixed. For example, female participants with higher HR reactivity to acute stressors show larger delay discounting (more temporal impulsivity), but this trend does not hold in males (Diller et al. 2011). These results indicate that the stress reactivity of the autonomic nervous system might be related to impulsivity. On the other hand, others do not find significant associations between HR and HR reactivity with delay discounting rates (e.g. Kimura et al. 2013). Instead, stress increases delay discounting only in individuals manifesting a cortisol increase, a putative biomarker of stress. A recent study (Lempert et al. 2017) using within-subjects design reported that blunting arousal levels by administration of the β-adrenergic receptor antagonist propranolol also did not affect temporal discounting rates. Together, these results indicate that the effects of arousal on delay discounting might not be straightforward and may mainly depend on individual changes in arousal level, which may affect biological changes in different ways.

### Probability discounting

In contrast to temporal discounting, we found no association between the change in arousal and probability discounting. Indeed, in rodents during risky decisions, yohimbine does not affect probabilistic discounting per se (Montes et al. 2015) but rather impairs the flexibility of response adjustments. Thus, when reward probabilities are initially large and then decrease (descending condition), yohimbine increases the number of risky choices in later blocks. The reverse is true for ascending condition (when the reward probabilities are initially small and then increase)—yohimbine results in reduced preference for riskier options. In our study, the trials of different probabilities were intermixed (there was no ascending/descending condition); hence, risky decisions were more likely to be tested. The observed lack of an effect of yohimbine in our task confirms the notion that noradrenergic activation may not directly impact risky decisions. These findings appear in contrast to the observation that increased physiological arousal following physical exercise is associated with less risky behaviour in a gambling task (Schmidt et al. 2013). However, we asked explicit hypothetical choices, in contrast to gambling game paradigms, in which the outcomes are real (Schmidt et al. 2013). It is plausible, therefore, that the type of risk task (hypothetical vs real) is differentially affected by arousal level. The same may apply to temporal discounting task which also included hypothetical decisions only. Future studies should assess the differences between the role of arousal on decision-making involving real versus hypothetical gains.

### Reflection impulsivity

To our knowledge, this is the first investigation of the role of physiological arousal mediated by noradrenergic mechanisms in reflection impulsivity. Our results suggest that the DIA BP reactivity negatively correlates with the degree of information sampling in the IST RC condition. Therefore, the results provide an indication that individuals showing a greater increase in DIA BP also gathered less information before deciding in the task. Importantly, this relationship was only present in the reward conflict condition, in which the potential gains decrease as participants sample more data (information sampling/reward trade-off), and not in the fixed win condition, in which gathering as much information as possible is the most advantageous strategy. Therefore, state arousal may affect reflection impulsivity in more challenging and more ambiguous circumstances.

## Conclusions

In conclusion, our findings indicate that yohimbine-induced arousal is associated with decreased motor impulsivity, suggesting that yohimbine treatment might prove us a means of reducing ‘stopping’ impulsivity. Moreover, increased arousal, at the individuals’ level, is associated with decreased temporal but increased reflection impulsivity. Probability discounting, a measure of risk taking, was not related to arousal level. These results further support the notion that distinct subtypes of impulsivity are differentially affected by modulators. Additionally, we did not find, in this normative sample, evidence for the under-arousal hypothesis of impulsivity (Barratt 1985; Eysenck and Eysenck 1985; Zuckerman 1969), since we did not observe any relationship between resting measures of arousal and trait impulsivity.

These data highlight the importance of state of physiological arousal in behavioural impulsivity.

## Electronic supplementary material

ESM 1 (PDF 241 kb)
