# Supplementary material for: The impact of Yohimbine-induced arousal on facets of behavioural impulsivity
Source: Psychopharmacology (Berl). 2019 Jan 11;236(6):1783–95. doi: 10.1007/s00213-018-5160-9 (PMC6602985; doi:10.1007/s00213-018-5160-9)
Supplement: Supplementary file 1 — (PDF 241 kb) [file 213_2018_5160_MOESM1_ESM.pdf]

Appendix

Article Title: The impact of Yohimbine-induced arousal on facets of behavioural impulsivity

Journal Name: *Psychopharmacology*

Authors and Affiliations: Aleksandra M. Herman[1,2]*, Hugo D. Critchley[3,4,5], Theodora Duka[1,2]

[1] Behavioural and Clinical Neuroscience, School of Psychology, University of Sussex, UK

[2] Sussex Addiction and Intervention Centre, University of Sussex, UK

[3] Sackler Centre for Consciousness Science, University of Sussex, UK

[4] Department of Neuroscience, Brighton & Sussex Medical School, UK

[5] Sussex Partnership NHS Foundation Trust, UK

*Corresponding author:

Address: School of Psychology, University of Sussex, Brighton, BN1 9QH, UK

E-mail: a.herman@sussex.ac.uk

Telephone: +44 01273 872803

Acknowledgements: The work was funded by the Sussex Neuroscience 4-year PhD studentship awarded to AH.

Supplementary Results and Discussion

We performed an additional set of analyses exploring sex differences in trait, physiological and behavioural measures. We report these analyses as they may be of interest for planning future experiments; however, we caution the readers against driving strong conclusions from these results as our study was not designed to examine sex differences in yohimbine-induced changes in impulsive behaviours and, therefore, is underpowered for this purpose.

To compare male and female participants on demographics, trait and physiological measures, independent samples *t*-test were performed. Overall, males and females showed no differences in any of the trait measures (Table A1). Males, however, were heavier and taller than females and showed elevated systolic blood pressure at baseline, relative to female participants (see Table A1 for details).

For behavioural data analysis, we employed 2 (Placebo vs Yohimbine) x 2 (Males vs Females) ANOVAs. As an exception, for analysis of the Affective Stop Signal Task (ASST) data, mixed ANOVA with emotion condition (neutral vs fearful) as a within-subject factor was employed. Descriptive statistics of behavioural variables is presented in Table A2.

ASST

The analysis of the stop signal reaction time (SSRT) revealed a three-way (Emotion x Group x Sex) interaction [$F(1,34) = 4.45$, $p = .042$, $n_p^2 = 0.12$; the interaction is also approaching significance when controlling for drug manipulation group differences in sensation seeking: $F(1,33) = 3.91$, $p = .057$, $n_p^2 = 0.11$]. Post-hoc repeated measures ANOVA, indicated that response inhibition in males, but not females, was differentially affected by emotional context depending on the drug manipulation (for details see Table A3). Specifically, males showed lower inhibitory control (higher SSRTs) in the fearful relative to the neutral context under placebo (the effect approaching significance following the Bonferroni correction for multiple

comparisons $\alpha \leq .025$); however, this effect was not present under yohimbine. There were no other significant effects or interactions (see Table A3 and Fig. A1 for details). Together, these results suggest that males were more affected by the task-irrelevant emotional context than females under placebo; however, these effects disappear under the influence of yohimbine.

The previous study by Schwabe et al. (2013) indicated that yohimbine induces opposite effects on fearfulness ratings of fearful facial expressions in males and females: Increasing fearfulness ratings in females while decreasing them in males, with no effect on neutral facial expressions ratings. These behavioural effects were related to enhanced amygdala activity for fearful faces in women, but decreased activity in men. Although we did not find an effect of yohimbine on response inhibition in females, our findings suggest that males might be less reactive to task-irrelevant emotional context under the influence of yohimbine than placebo, partially corroborating findings by Schwabe and colleagues.

MCQ

A main effect of sex on the proportion of larger delayed rewards (LDR) was found [$F(1,36) = 4.55$, $p = .040$, $n_p^2 = 0.11$], indicating that females showed lower temporal impulsivity than males regardless of the drug manipulation (Fig. A2). There were no other significant results [main effect of drug: $F(1,36) = 1.45$, $p = .236$, $n_p^2 = 0.03$; interaction: $F(1,36) = 0.17$, $p = .166$, $n_p^2 < 0.01$].

Overall, these findings corroborate the past literature suggesting that males present higher temporal impulsivity than females (Silverman 2003; Herman et al. 2018).

PD and IST

There were no significant main effects or an interaction effect related to the performance on the Probability Discounting and Information Sampling Task (*F*'s < 1.92, *p*'s > .17, n_p^2's < 0.05), indicating that there were no sex differences in the performance on the task and that pharmacological manipulation did not affect the performance differently in males and females.

Table A1 Sex differences in demographical information, trait and physiological measures.

Variable		Males				Females				Independent Samples *t*-Test			
		N	Mean	SD	SE	N	Mean	SD	SE	*t*	df	*p*	Cohen's d
**Demographic information**													
Age		19	23.42	5.30	1.22	23	21.26	3.60	0.75	1.57	40	0.125	0.49
Weight [kg]		19	75.45	8.86	2.03	23	65.45	7.43	1.55	**3.98**	**40**	**< .001**	**1.24**
Height [m]		19	1.82	0.07	0.02	23	1.69	0.07	0.02	**6.20**	**40**	**< .001**	**1.92**
BMI [kg/m²]		19	22.84	2.41	0.55	23	23.06	2.62	0.55	0.28	40	0.783	-0.09
Alcohol Units per week		19	14.06	12.76	2.93	23	10.58	9.15	1.91	1.03	40	0.311	0.32
RAVLT		16	5.69	1.70	0.43	21	6.62	1.83	0.40	1.58	35	0.123	-0.53
**Trait impulsivity**													
BIS Total		19	64.42	9.37	2.15	23	65.22	11.25	2.35	0.25	40	0.807	-0.08
UPPS-P	Negative Urgency	19	25.21	6.10	1.40	23	28.57	6.06	1.26	1.78	40	0.083	-0.55
	Premeditation	19	21.53	4.16	0.95	23	20.87	5.66	1.18	0.42	40	0.676	0.13
	Perseverance	19	19.95	4.59	1.05	23	19.91	5.66	1.18	0.02	40	0.983	0.01
	Sensation Seeking	19	36.68	8.25	1.89	23	38.00	5.93	1.24	0.60	40	0.552	-0.19
	Positive Urgency	19	27.95	8.63	1.98	23	28.91	9.42	1.97	0.34	40	0.733	-0.11
**Mood measures**													
PANAS	NA Pre	19	12.11	2.36	0.54	23	12.44	2.63	0.55	0.42	40	0.674	-0.13
	PA Pre	19	30.21	7.44	1.71	23	28.65	6.08	1.27	0.75	40	0.459	0.23
STAI	Trait Anxiety	19	38.95	7.31	1.68	23	39.78	7.23	1.51	0.37	40	0.713	-0.12
	State Anxiety	19	33.58	6.65	1.53	23	34.00	8.83	1.84	0.17	40	0.865	-0.05
**Physiological measures**													
Baseline SYS BP		19	119.26	10.86	2.49	23	107.70	8.29	1.73	**3.91**	**40**	**< .001**	**1.21**
Baseline DIA BP		19	72.42	8.86	2.03	23	71.39	5.88	1.23	0.45	40	0.655	0.14
Baseline HR		19	66.53	9.90	2.27	23	69.52	7.12	1.48	1.14	40	0.261	-0.35

Table A2 Performance on the behavioural tasks by drug manipulation group for males and females.

Variables		Sex	Placebo			Yohimbine		
			N	Mean	SD	N	Mean	SD
IST	RC P(correct)	Male	9	0.75	0.08	10	0.71	0.09
		Female	12	0.72	0.05	11	0.72	0.10
	FW P(correct)	Male	9	0.84	0.10	10	0.80	0.09
		Female	12	0.77	0.06	11	0.82	0.14
PD	ln(h)	Male	9	0.51	0.99	10	0.75	0.72
		Female	12	0.61	0.84	11	0.33	0.78
MCQ	Proportion LDR	Male	8	0.34	0.08	10	0.43	0.26
		Female	11	0.49	0.18	11	0.54	0.17
SST	Neutral SSRT	Male	8	272.80	55.00	9	287.80	35.81
		Female	11	308.50	59.27	10	273.60	46.92
	Fearful SSRT	Male	8	322.90	75.73	9	277.60	31.84
		Female	11	307.40	64.72	10	282.00	44.72

Table A3 Results from the Affective Stop Signal Task analysis.

Mixed ANOVA

Within Subjects Effects		Sum of Squares	df	Mean Square	F	p	η^2_p
Emotion		2604.00	1	2604.00	2.03	0.164	0.06
Emotion ✻ Drug		3031.00	1	3031.00	2.36	0.134	0.07
Emotion ✻ Sex		1245.00	1	1245.00	0.97	0.332	0.03
Emotion ✻ Drug ✻ Sex		5708.00	1	5708.00	4.45	**0.042**	0.12
Residual		43648.00	34	1284.00			
**Between Subjects Effects**							
Drug		9619.30	1	9619.30	2.17	0.150	0.06
Sex		129.50	1	129.50	0.03	0.865	0.00
Drug ✻ Sex		1045.90	1	1045.90	0.24	0.630	0.01
Residual		150862.60	34	4437.10			
**Post-hoc tests: Repeated measures ANOVA**							
Male	Emotion	6735.69	1	6735.69	2.53	0.133 *	0.14
	Emotion ✻ Drug	15425.13	1	15425.13	5.78	0.030 *	0.28
	Residual	40009.19	15	2667.28			
Female	Emotion	277.27	1	277.27	0.11	0.742 *	0.01
	Emotion ✻ Drug	469.85	1	469.85	0.19	0.669 *	0.01
	Residual	47285.91	19	2488.73			

Note. Type III Sum of Squares; *uncorrected p-value.

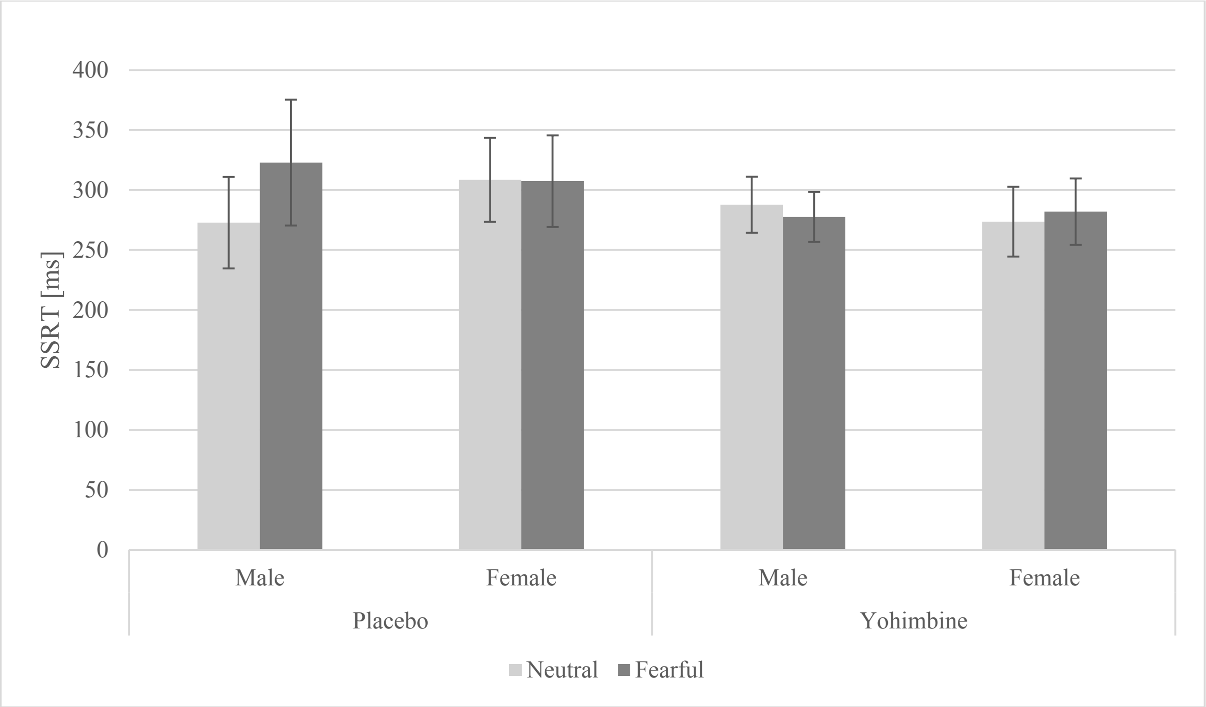

Fig. A1 Three-way (Group by Emotion by Sex) interaction effect on response inhibition on the Affective Stop Signal Task. Error bars represent 95% confidence intervals. SSRT – Stop signal Reaction Time

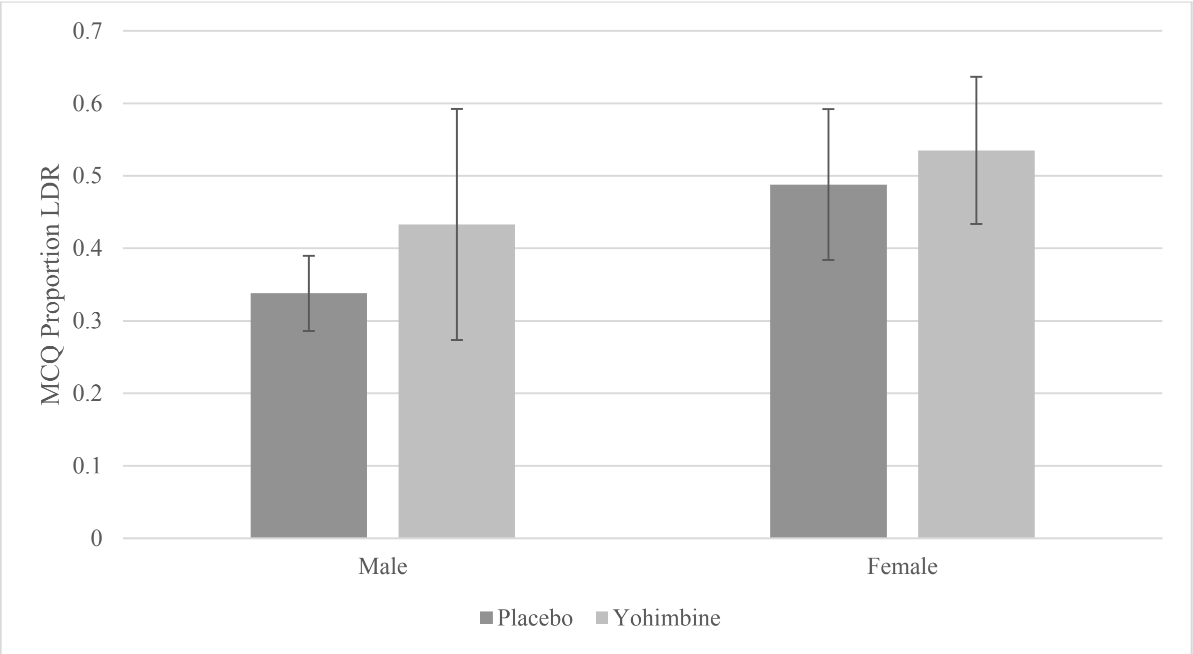

Fig. A2 Proportion of larger delayed rewards (LDR) selected in the Monetary Choice Questionnaire (MCQ) by drug manipulation group for males and females. Error bars represent 95% Confidence Intervals.

References:

Herman AM, Critchley HD, Duka T (2018) Risk-taking and impulsivity: The role of mood states and interoception. Front Psychol 9:. doi: 10.3389/fpsyg.2018.01625

Schwabe L, Höffken O, Tegenthoff M, Wolf OT (2013) Opposite effects of noradrenergic arousal on amygdala processing of fearful faces in men and women. Neuroimage 73:1–7. doi: 10.1016/j.neuroimage.2013.01.057

Silverman IW (2003) Gender Differences in Delay of Gratification: A Meta-Analysis. Sex Roles 49:451–463. doi: 10.1023/A:1025872421115
